# Measurement of Circulating 25-Hydroxy Vitamin D Using Three Commercial Enzyme-Linked Immunosorbent Assay Kits with Comparison to Liquid Chromatography: Tandem Mass Spectrometry Method

**DOI:** 10.5402/2013/723139

**Published:** 2013-08-13

**Authors:** Cheng-Shiun He, Michael Gleeson, William D. Fraser

**Affiliations:** ^1^School of Sport, Exercise and Health Sciences, Loughborough University, Leicestershire, Loughborough LE11 3TU, UK; ^2^Norwich Medical School, University of East Anglia, Norwich NR4 7TJ, UK

## Abstract

*Aim.* The purpose of this study was to compare the accuracy and clinical implications of three commercial enzyme-linked immunosorbent assay (ELISA) kits (Eagle Biosciences, Immundiagnostik, and MicroVue) with a validated liquid chromatography-tandem mass spectrometry (LC-MS/MS) method for the measurement of serum 25(OH)D concentration. *Methods.* Blood samples were obtained from 225 healthy individuals who were recruited as subjects from Loughborough University, UK. Plasma samples were measured for 25(OH)D concentration by means of LC-MS/MS and ELISA kits from Eagle Biosciences, Immundiagnostik, and MicroVue. *Results.* The 25(OH)D concentration measured by the Eagle Biosciences, Immundiagnostik, and MicroVue ELISAs biased −50.9 ± 79.1 nmol/L, −14.2 ± 91.0 nmol/L, and −7.2 ± 18.9 nmol/L (bias ± SD) from the LC-MS/MS method, respectively. We found that 52% (Eagle Biosciences), 48% (Immundiagnostik), and 38% (MicroVue) of participants were misclassified, and the results showed the poor agreement (Kappa: −0.201~0.251) in classification of participants defined as vitamin D sufficiency and insufficiency between each method and LC-MS/MS. *Conclusions.* The present study demonstrated that there were negative biases and considerable misclassification of participants using the cut-off point (50 nmol/L) for vitamin D insufficiency and sufficiency using the Eagle Biosciences, Immundiagnostik, and MicroVue ELISAs compared with the LC-MS/MS assay.

## 1. Introduction

Recently, there have been growing demands for measurement of vitamin D status because of the high prevalence of vitamin D insufficiency and the discovery of vitamin D nonclassical functions [1, 2]. The high prevalence of vitamin D insufficiency in the general population worldwide has been documented in a large number of studies [3]. Moreover, vitamin D insufficiency has also been reported to be common in athletes especially if exposure to natural sunlight is limited (e.g., when training in the winter months or when training mostly indoors) [4]. In addition, it has been recently recognised that vitamin D plays an important role in upregulating immunity. Several recent studies have found a negative association between vitamin D status and respiratory illness incidence in young and elderly adults [5, 6].

Measurement of plasma 25-hydroxy vitamin D (25(OH)D) concentration is widely used in clinical practice and research reports to assess vitamin D status. In humans, vitamin D can be obtained either from dietary sources or the epidermal layer of the skin via exposure to sunlight. Two forms of vitamin D can be obtained from dietary sources: vitamin D3 (cholecalciferol) and vitamin D2 (ergocalciferol). While vitamin D3 is found in food from animal origin, such as cod-liver oil, salmon, and egg yolk, vitamin D2 is present in some plants and fungi. The endogenously synthesised vitamin D3 and diet-derived D2 and D3 must be hydroxylated in the liver into 25(OH)D. 25(OH)D is the main storage form, which can be stored in muscles and adipose tissue and is the major circulating metabolite of vitamin D, with a plasma half-life of 2-3 weeks. Therefore, the plasma concentration of 25(OH)D is considered to be the primary indicator of vitamin D status [7]. Plasma 25(OH)D values commonly accepted as the reference range [8] are as follows. In healthy humans, 25(OH)D plasma levels > 100 nmol/L are defined as optimal vitamin D status and levels from 50 to 100 nmol/L are defined as adequate. Serum levels of 25(OH)D < 50 nmol/L are proposed to define inadequate vitamin D status, and values <30 nmol/L represent vitamin D deficiency.

Plasma 25(OH)D concentration can be measured by competitive protein binding assay, immunoassay, HPLC, and LC-MS/MS [1]. The LC-MS/MS method is generally considered to be the best way for the measurement of serum 25(OH)D levels because it can separate and accurately quantitate both 25(OH)D_2_ and 25(OH)D_3_ [2, 9]; furthermore, an extraction procedure ensures that both free 25(OH)D and protein-bound 25(OH)D are quantified. However, as the LC-MS/MS method requires expensive equipment, large plasma sample volume, and specialised staff, the commercial ELISAs are the most popular method for the measurement of plasma 25(OH)D concentration [1, 2]. Current 25(OH)D ELISAs employ polyclonal or monoclonal antibodies that bind specifically to human 25(OH)D. However, the competition between the 25(OH)D specific antibody and vitamin D binding protein (VDBP) in plasma samples makes these assays difficult to control and several of the most commonly used commercial ELISA kits, such as those manufactured by DiaSorin, Immunodiagnostic Systems (IDS), and Roche, have been shown to agree only poorly with LC-MS/MS [1, 2, 10–12]. Given the recent explosion of interest in vitamin D, it seems to be necessary to establish which of the commercial ELISAs for plasma/serum 25(OH)D are the most reliable. The obvious potential advantages of these methods are their relative ease of use, low cost, and high throughput using small plasma sample volumes.

The aim of this study was to compare the accuracy and clinical implications of three commercial ELISA kits (Eagle Biosciences, Immundiagnostik, and MicroVue) with LC-MS/MS method (carried out in a validated laboratory) for the measurement of plasma 25(OH)D concentration.

## 2. Methods

### 2.1. Participants

A total of 225 healthy individuals were recruited as subjects from Loughborough University, UK (latitude 53°N) during November 2011, in our previous vitamin D study with the mean age of the study cohort at recruitment being 21 ± 3 years (mean ± SD). Subjects were required to complete a comprehensive health-screening questionnaire prior to starting the study and had not taken any regular medication or antibiotics in the last 3 months prior to the study. All subjects were fully informed about the rationale for the study and of all the experimental procedures to be undertaken. Subjects provided written consent to participate in the study, which had earlier received the approval of Loughborough University Ethical Advisory Committee. For the visit to the laboratory, subjects arrived in the morning at 08:30–10:30 following an overnight fast of approximately 12 hrs, and their body mass and height were recorded. Information about the study was given to them, and they then signed an informed consent form. Subsequently, a resting venous blood sample (12 mL) was obtained by venipuncture from an antecubital forearm vein into the vacutainer tube (Becton Dickinson, Oxford, UK) containing K_3_EDTA. Haematological analysis was immediately carried out on the EDTA sample (including haemoglobin, haematocrit and total and differential leukocyte counts) using an automated cell-counter (Ac.T 5diff haematology analyser, Beckman Coulter, High Wycombe, UK). Subjects had to have normal haematology to be included in the study. The remaining EDTA blood was centrifuged for 10 min at 1500 g and 4°C, and the plasma was stored at −80°C prior to analysis. Plasma samples were measured for 25(OH)D concentration by means of LC-MS/MS and subsequently by ELISA kits from Eagle Biosciences (Nashua, NH, USA), Immundiagnostik (Bensheim and Biomedica, Vienna, Austria), and MicroVue (Hannover, Germany).

### 2.2. Liquid Chromatography: Tandem Mass Spectrometry

In our previous vitamin D study, 225 EDTA plasma samples were analysed for 25(OH)D_3_, and 25(OH)D_2_ with a high pressure liquid chromatography tandem mass spectrometer (Waters Acuity, Manchester, UK) after a maximum of 10 months in storage with no previous freeze-thaw cycles as described previously [13]. Briefly, 25(OH)D_2_, 25(OH)D_3_ and deuterated internal standard were extracted from plasma samples, following protein precipitation, using isolute C18 solid phase extraction cartridges. Potential interfering compounds were removed by initial elution with 50% methanol followed by elution of the vitamins using 10% tetrahydrofuran in acetonitrile. Dried extracts were reconstituted prior to injection into a high performance liquid chromatography tandem mass spectrometer in the multiple reaction mode (MRM). The MRM transitions (*m/z*) used were 413.2 > 395.3, 401.1 > 383.3, and 407.5 > 107.2 for 25(OH)D_2_, 25(OH)D_3_ and hexadeuterated (OH)D_3_ (internal standard), respectively. Intraassay CVs were <10% across a working range of 2.5–624 nmol/L for both 25(OH)D_3_ and 25(OH)D_2_. Measurements were performed in a laboratory (Norwich University Hospital, Norwich, UK) meeting the performance target set by the Vitamin D External Quality Assessment Scheme (DEQAS) Advisory Panel for 25(OH)D assays.

### 2.3. Eagle Biosciences

29 randomly selected EDTA plasma samples with sufficient volume were assayed for 25(OH)D concentration using a commercially available ELISA kit (Eagle Biosciences, Nashua, NH, USA) according to the manufacturer's instructions. Briefly, the calibrators and patient samples are diluted with biotin-labelled 25(OH)D and added to microplate wells coated with monoclonal anti-25(OH)D antibodies in the first analysis step. An unknown amount of 25(OH)D in the patient sample and a known amount of biotin-labelled 25(OH)D compete for the antibody binding sites in the microplate wells plate during the incubation. Unbound 25(OH)D is removed by washing. For the detection of bound biotin-labelled 25(OH)D, a second incubation is performed using peroxidase-labelled streptavidin. In a third incubation using the peroxidase substrate tetramethylbenzidine (TMB), the bound peroxidase promotes a colour reaction. An acidic stopping solution is then added to stop the reaction. The colour intensity is inversely proportional to the 25(OH)D concentration. According to information supplied by the manufacturer, the intraassay CVs were 4.9% at a 25(OH)D mean concentration of 27.0 nmol/L, 6.9% at a 25(OH)D mean concentration of 61.5 nmol/L and 3.2% at a 25(OH)D mean concentration of 160.3 nmol/L, respectively.

### 2.4. Immundiagnostik

29 randomly selected EDTA plasma samples with sufficient volume were assayed for 25(OH)D concentration using a commercially available ELISA kit (Immundiagnostik, Bensheim and Biomedica, Vienna, Austria) according to the manufacturer's instructions. Briefly, in the first incubation step, sample, calibrator, control, the vitamin D binding protein (VDBP) and the VDBP-Antibody are added to the solid phase. 25(OH)D present in the sample then competes with the tracer, coated on the well for the specific binding site of the binding protein, and the VDBP antibody is bound to the vitamin binding protein. Hence, with increasing concentrations of 25(OH)D in the sample, the amount of binding protein immobilized to the well via the tracer is reduced. After a washing step to remove unbound components, the quantification of VDBP is achieved by incubation with a host specific peroxidase labelled antibody using TMB as enzyme substrate. An acidic stopping solution is then added to stop the reaction, and the colour converts to yellow. The intensity of the yellow colour is indirectly proportional to the concentration of 25(OH)D in the sample. The detection limit of the assay was 5.6 nmol/L. According to the information supplied by the manufacturer, the mean intraassay and inter-assay CVs of the 25(OH)D assay were 10.7% and 11.8% to 13.2%, respectively.

### 2.5. MicroVue

37 randomly selected EDTA plasma samples with sufficient volume were assayed for 25(OH)D concentration using a commercially available immunoassay kit (MicroVue, Hannover, Germany) according to the manufacturer's instructions. Briefly, in step 1, standards, controls, and test specimens are added to microplate wells precoated with a primary monoclonal anti-human 25(OH)D_2_ and 25(OH)D_3_ antibody. Total 25(OH)D (D_2_ and D_3_) in the standards, controls, and samples is dissociated from serum binding proteins and binds to the monoclonal antibody. In step 2, following the first wash cycle, a fixed amount of biotinylated 25(OH)D, in the presence of streptavidin-horseradish peroxidase (HRP), competes with the unlabelled 25(OH)D_2_ and 25(OH)D_3_ bound to the monoclonal antibody. At the end of the assay incubation period, a wash cycle stops the competition reaction. In step 3, a chromogenic enzyme substrate is added to each microplate well. The bound HRP conjugate reacts with the substrate, forming a blue colour. After incubation, the enzyme reaction is terminated using a stop chemical, and the colour changes to yellow. The colour intensity of the reaction mixture is proportional to the concentration of total 25(OH)D present in the test specimens, standards, and controls. According to the information supplied by the manufacturer, the intraassay CVs were 5.7% at a 25(OH)D mean concentration of 68.5 nmol/L and 2.7% at a 25(OH)D mean concentration of 107.5 nmol/L, respectively.

### 2.6. Statistical Analysis

The Wilcoxon matched-pairs signed-ranks test was used to test for the differences between each method and LC-MS/MS. The correlation between each method and LC-MS/MS was compared using Pearson's correlation coefficient. Agreement in classification of results (vitamin D sufficiency: ≥50 nmol/L; vitamin D insufficiency: <50 nmol/L) between each method and LC-MS/MS was assessed using Cohen's kappa (agreement: <0, no; 0–0.4, poor; 0.4–0.75, fair to good; and >0.75, excellent) [14]. The simple linear regression and Bland-Altman plots were used for the comparison of each method and LC-MS/MS. Data are presented as mean (±SD), and the accepted level of significance was *P* < 0.05.

## 3. Results

### 3.1. LC-MS/MS and Eagle Biosciences

**Figure 1:**
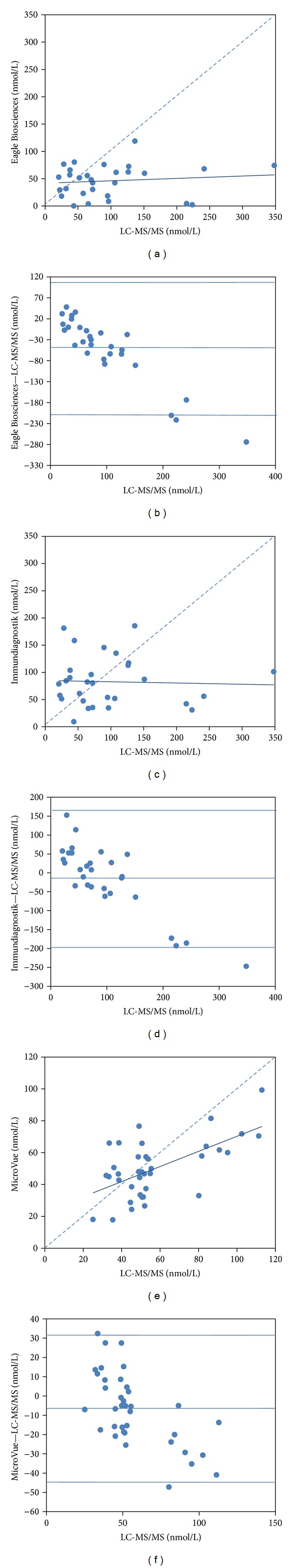
Comparison of the LC-MS/MS method with ELISAs from Eagle Biosciences ((a), (b)), Immundiagnostik ((c), (d)), and MicroVue ((e), (f)) by the simple linear regression ((a), (c), and (e)) and Bland-Altman plots ((b), (d), and (f)).

The 25(OH)D concentration measured by the Eagle Biosciences assay was significantly lower than the LC-MS/MS assay (Eagle Biosciences: 46.1 ± 29.0 nmol/L, LC-MS/MS: 97.0 ± 77.0 nmol/L; *P* = 0.001) and biased −50.9 ± 79.1 nmol/L (bias ± SD; 95% limits of agreement: −209.1, 107.3) from the LC-MS/MS assay (Table 1 and Figure 1(b)). There was no significant correlation (*r* = 0.115, *P* = 0.551) between the LC-MS/MS assay and the Eagle Biosciences assay (Table 1). Moreover, there was a considerable misclassification of participants using the cut-off point for vitamin D insufficiency and sufficiency between these two assays. The data of our study showed that 52% (15/29) of participants were misclassified when the results from the Eagle Biosciences assay were compared with those from the LC-MS/MS assay. In addition, there were 14% (4/29) of participants classified as vitamin D insufficient according to the results from both assays. Cohen's kappa coefficient was −0.048 indicating no agreement (Table 2(a)).

### 3.2. LC-MS/MS and Immundiagnostik

There was no significant difference in the 25(OH)D concentration between the Immundiagnostik assay and the LC-MS/MS assay (Immundiagnostik: 82.8 ± 45.7 nmol/L, LC-MS/MS: 97.0 ± 77.0 nmol/L; *P* = 0.770) (Table 1). The Immundiagnostik assay biased −14.2 ± 91.0 nmol/L (bias ± SD; 95% limits of agreement: −196.3, 167.9) from the LC-MS/MS assay (Figure 1(d)). However, there was no significantly correlation between the LC-MS/MS assay and the Immundiagnostik assay (*r* = −0.039, *P* = 0.843) (Table 1). The data of this study showed that 48% (14/29) of participants were misclassified when the results from the Immundiagnostik assay were compared with those from the LC-MS/MS assay. In addition, there were only 3% (1/29) of participants classified as vitamin D insufficient according to the results from both assays. Cohen's kappa coefficient was −0.201 indicating no agreement (Table 2(b)).

### 3.3. LC-MS/MS and MicroVue

The 25(OH)D concentration measured by the MicroVue assay was significantly lower than the LC-MS/MS assay (MicroVue: 50.0 ± 18.1 nmol/L; LC-MS/MS: 57.1 ± 23.1 nmol/L, *P* = 0.023) and biased −7.2 ± 18.9 nmol/L (bias ± SD; 95% limits of agreement: −44.9, 30.6) from the LC-MS/MS assay (Table 1 and Figure 1(f)). Nevertheless, there was a significant positive correlation between the LC-MS/MS assay and the MicroVue assay (*r* = 0.603, *P* = 0.001) (Table 1). The data of this study showed that 38% (9/37) of participants were misclassified when the results from the MicroVue assay were compared with those from the LC-MS/MS assay. Moreover, there were 32% (12/37) of participants classified as vitamin D insufficient according the results from both assays. Cohen's kappa coefficient was 0.251 indicating poor agreement (Table 2(c)).

## 4. Discussion

To our knowledge, this is the first report to compare the accuracy and clinical implications of the Eagle Biosciences, Immundiagnostik, and MicroVue commercial immunoassay kits with the LC-MS/MS method for the measurement of plasma 25(OH)D concentration. The main findings were that there were negative biases in the Eagle Biosciences, Immundiagnostik, and MicroVue assays compared with the LC-MS/MS assay. Furthermore, there was no significant correlation between Eagle Biosciences and LC-MS/MS as well as Immundiagnostik and LC-MS/MS. Nevertheless, there was a positive correlation between the MicroVue and LC-MS/MS assay. In addition, there was a considerable misclassification of participants using the cut-off point for vitamin D insufficiency and sufficiency between each assay and the LC-MS/MS assay. The results of the present study showed no agreement in classification of participants defined as vitamin D sufficient and insufficient between Eagle Biosciences and LC-MS/MS as well as Immundiagnostik and LC-MS/MS and only poor agreement between MicroVue and LC-MS/MS.

The reasons for the negative biases in the Eagle Biosciences, Immundiagnostik, and MicroVue assays compared with the LC-MS/MS assay are not clear. However, a confounding factor for the variable results might be due to the strong binding of 25(OH)D to VDBP. The serum 25(OH)D concentration cannot be measured accurately unless it is released from VDBP and the strong protein binding of 25(OH)D requires the employment of suitable conditions to release 25(OH)D from VDBP [1, 2]. In the LC-MS/MS assay of the present study, 25(OH)D_2_ and 25(OH)D_3_ were extracted from plasma samples using isolute C18 solid phase extraction cartridges. Nevertheless, all the immunoassays that were examined in this study employed monoclonal 25(OH)D antibodies to bind 25(OH)D from VDBP. The competition between the specific antibody and VDBP in plasma samples could make these immunoassays difficult to control and may lead to poor agreement with the LC-MS/MS assay [10, 15]. Another confounding factor might be due to the quantitation of plasma 25(OH)D_2_ and 25(OH)D_3_. Because isotope dilution LC-MS/MS methods can simultaneously and accurately quantitate both 25(OH)D_2_ and 25(OH)D_3_, it can be considered to be the gold standard method for the measurement of plasma or serum 25(OH)D levels [1, 2, 9]. The present immunoassays cannot measure the concentration of 25(OH)D_2_ and 25(OH)D_3_ independently. It has been reported that there was an underestimation of plasma 25(OH)D_2_ concentration in several commercial immunoassays which resulted in marked variations of the total plasma 25(OH)D levels [1, 2]. Nevertheless, the results of the present study showed that there was a positively correlation between the MicroVue ELISA and LC-MS/MS methods and had better agreement than the other two commercial ELISAs that were examined. It might be due to the employment of both monoclonal anti-human 25(OH)D_2_ and 25(OH)D_3_ antibody in the MicroVue assay.

There was a considerable misclassification of participants using the cut-off point for vitamin D insufficiency and sufficiency in the Eagle Biosciences, Immundiagnostik, and MicroVue assays compared with the LC-MS/MS assay. In healthy humans, plasma 25(OH)D serum levels >100 nmol/L are defined as optimal vitamin D status, and levels from 50 to 100 nmol/L are defined as adequate. Serum levels of 25(OH)D <50 nmol/L are proposed to define inadequate vitamin D status, and values <30 nmol/L represent vitamin D deficiency [8]. Therefore, the commonly used cut-off point for vitamin D insufficiency in clinical practice and research reports is the threshold concentration of 25(OH)D of <50 nmol/L [11]. On the basis of the present data, we found that 52%, 48%, and 38% of participants were misclassified when the results from the Eagle Biosciences, Immundiagnostik and MicroVue assays were compared with those from the LC-MS/MS assay, respectively. In addition, according to Cohen's kappa coefficient from this study (kappa: −0.201~ 0.251), the results showed the poor agreement in classification of participants defined as vitamin D sufficient and insufficient between each method and LC-MS/MS [14]. Measurement of plasma 25(OH)D concentration is widely used in clinical practice and research reports to assess vitamin D status of participants. Our results indicated that the assessment of vitamin D status seems to be influenced substantively by the 25(OH)D assay being used. Given the large proportion of misclassified participants using the present immunoassays, it is important to discover if there are any more reliable commercial immunoassays of serum 25(OH)D, which are suitable for clinicians and researchers.

In conclusion, the present study demonstrated that there were negative biases and the considerable misclassification of participants using the cut-off point for vitamin D insufficiency and sufficiency using the Eagle Biosciences, Immundiagnostik, and MicroVue assays compared with the LC-MS/MS assay. Without using the most reliable 25(OH)D assays, the assessment of vitamin D status of participants remains very doubtful.

## Figures and Tables

**Table 1:** Comparison of mean (SD) 25(OH)D concentrations (nmol/L) from different 25(OH)D assays.

Sample size for comparison bloods	LC-MS/MS	Eagle Biosciences	Immundiagnostik	MicroVue	Wilcoxon signed-rank test	Pearson's correlation
*n* = 29	97.0 ± 77.0	46.1 ± 29.0			*P* = .001	*P* = .551
*n* = 29	97.0 ± 77.0		82.8 ± 45.7		*P* = .770	*P* = .843
*n* = 37	57.1 ± 23.1			50.0 ± 18.1	*P* = .023	*P* = .001

**Table:** (a)

*n* = 29	Eagle Biosciences	
	<50 nmol/L	≥50 nmol/L
LC-MS/MS		
<50 nmol/L	4 (14%)	5 (18%)
≥50 nmol/L	10 (34%)	10 (34%)
Cohen's kappa	−0.048	

**Table:** (b)

*n* = 29	Immundiagnostik	
	<50 nmol/L	≥50 nmol/L
LC-MS/MS		
<50 nmol/L	1 (3%)	8 (28%)
≥50 nmol/L	6 (21%)	14 (48%)
Cohen's kappa	−0.201	

**Table:** (c)

*n* = 37	MicroVue	
	<50 nmol/L	≥50 nmol/L
LC-MS/MS		
<50 nmol/L	12 (32%)	5 (14%)
≥50 nmol/L	9 (24%)	11 (30%)
Cohen's kappa	0.251	
